# Microalgae biotechnology in Nordic countries – the potential of local strains

**DOI:** 10.1111/ppl.12951

**Published:** 2019-03-25

**Authors:** Otilia Cheregi, Susanne Ekendahl, Johan Engelbrektsson, Niklas Strömberg, Anna Godhe, Cornelia Spetea

**Affiliations:** ^1^ Department of Biological and Environmental Sciences University of Gothenburg Gothenburg 40530 Sweden; ^2^ Department of Chemistry and Materials RISE Research Institutes of Sweden Borås 50115 Sweden; ^3^ Department of Marine Sciences University of Gothenburg Gothenburg 40530 Sweden

## Abstract

Climate change, energy use and food security are the main challenges that our society is facing nowadays. Biofuels and feedstock from microalgae can be part of the solution if high and continuous production is to be ensured. This could be attained in year‐round, low cost, outdoor cultivation systems using strains that are not only champion producers of desired compounds but also have robust growth in a dynamic climate. Using microalgae strains adapted to the local conditions may be advantageous particularly in Nordic countries. Here, we review the current status of laboratory and outdoor‐scale cultivation in Nordic conditions of local strains for biofuel, high‐value compounds and water remediation. Strains suitable for biotechnological purposes were identified from the large and diverse pool represented by saline (NE Atlantic Ocean), brackish (Baltic Sea) and fresh water (lakes and rivers) sources. Energy‐efficient annual rotation for cultivation of strains well adapted to Nordic climate has the potential to provide high biomass yields for biotechnological purposes.

**Abbreviations**
EPA: eicosapentaenoic acid
DHA: docosahexaenoic acid
NPQ: non‐photochemical quenching
PSII: photosystem II
PUFA: polyunsaturated fatty acids
TAG: triacylglycerol

## Introduction

The recent meeting of the International Panel on Climate Change has announced that the global temperature has increased 1°C relative to preindustrial levels (IPCC 2018). To limit the global warming by 1.5°C, IPCC has urged governments to cut greenhouse gas emissions to at least half of the 2017 levels by 2030 and to achieve carbon neutrality by 2050. Without doubt, energy sources that are carbon neutral and renewable are urgently needed as alternatives to fossil fuels. Among them, hydroelectric, solar, wind, geothermal power and biofuels are already used to reduce the consumption of fossil fuels. Significant opportunities for biofuel production come from the cultivation of microalgae, which are aquatic microscopic organisms able to use light, water, nutrients and CO_2_ to produce biomass in a similar way as in plant photosynthesis. Properties that make microalgae superior to classical energy crops (e.g. rapeseed, soy, corn) are the following: (1) 5–20‐fold more productive per ha basis; (2) can grow on non‐arable land and are able to use various water sources (fresh, saline, waste); (3) allow multiple harvests because they double biomass in 24 h and can be produced throughout the year; (4) store lipids, mainly as triacylglycerols (TAGs), which are readily available for biodiesel production; (5) reduce greenhouse gas emissions, especially when using exhaust CO_2_ (Rodolfi et al. 2009, Weyer et al. 2010). The advantages of microalgae cultivation could potentially go beyond solving the energy challenge. Depending on the species and on the cultivation conditions, they are also a sustainable source of high‐value compounds such as proteins, pigments, antioxidants, polyunsaturated fatty acids (PUFA) and bioactive compounds (Maeda et al. 2018).

Most microalgae strains are cultivated at 20–35°C, which is generally accepted as the optimal temperature range for growth. Both higher and lower temperatures negatively affect microalgae growth; therefore, temperature control could be a reasonable strategy to preserve high growth rates. However, cooling or heating of a cultivation system adds to the price of the final product. The success of microalgae as feedstock depends on achieving high yields of biomass with minimum cost. Therefore, microalgae must be cultivated outdoors on areas in the scale of millions of ha, as humanity has been doing with agricultural crops since centuries. Cultivation in large outdoor facilities means limited control over environmental conditions, emphasizing the importance of selecting species/strains that are flexible and robust. In this review, we will focus on the selection and use in biotechnology of microalgae strains adapted at temperatures ranging between 4 and 20°C, specific for Nordic countries (Denmark, Sweden, Finland, Norway and Iceland). The geographic location of Nordic countries covers two large masses of water: the saline waters of the NE Atlantic (including the North Sea) and the brackish waters of the Baltic Sea. Although apparently not the ideal place for large scale cultivation, microalgae could be part of the solution for sustainable energy and food production in Nordic countries. A global evaluation of biofuel production from microalgae using a validated outdoor photobioreactor shows that even Sweden can supplement 30% of the transportation fuel consumption by cultivating microalgae on 20% of the non‐arable land (Moody et al. 2014).

Below we review the characteristic climate in Nordic countries and the way it impacts the strategies for microalgae use in biotechnology, with examples of recent studies using local strains and/or local conditions. Although ‘microalgae’ may include photosynthetic prokaryotes (cyanobacteria), our focus is on studies using eukaryotic microalgae.

## The climate of Northern Europe, photosynthesis and adaptation of microalgae

The five Nordic countries span the geographic area between 40 and 80°N and include zones with continental, subarctic and artic climate. The continental climate is milder than that of other countries at the same latitude (e.g. continental Canada, Russia) due to vicinity of the ocean and the effect of Gulf Stream bringing warm waters from the coast of Florida.

Photosynthesis by phytoplankton is the main source of primary production on Earth accounting for 50% of global CO_2_ fixation (50 Gt C year^−1^) (Falkowski and Wilson 1992). The major environmental factors that control photosynthesis and algal growth are light, temperature and the availability of nutrients. While light affects mainly photosynthesis, temperature affects all molecular activities and hence, the metabolism. The combination of these two factors and the availability of nutrients, controls the primary production in areas located at mid and high latitude. Growth of microalgae is usually limited in these areas by the low light intensity, short photoperiod and the low water temperatures. In the more Northern regions (at latitudes higher than 66°N, north of the Polar Circle), algal growth is affected by the prolonged darkness during the Nordic winter and, in some regions, by ice coverage.

Based on the geographic distribution with distinct temperature and light environments, microalgae are classified in polar, temperate and tropical species. However, above the Polar Circle, the sun stays above the horizon for the whole 24 hours (polar summer) while during the polar winter, the sun is under the horizon for the entire duration of a day. For this particular area, light is the most important factor that controls the distribution and seasonal occurrence of various species (Berge et al. 2015), while survival in prolonged darkness is an essential feature of polar microalgae (Peters and Thomas 1996). Microalgae isolated from both the north and the south pole are able to recover after 3–9 months in complete darkness through entering a physiologically dormant state, when cell division is stopped, respiration is at minimum and the chloroplast is reorganized by forming structures suitable for storage of products (Baldisserotto et al. 2005). Although at latitudes below the polar circle, light is still available, temperate microalgae might still experience prolonged darkness periods due to mixing into the aphotic zone. Compared to polar species, the survival of algae from the temperate regions in prolonged darkness is restricted to shorter periods, between 1 and 3 months (Peters 1996). The inverse correlation between temperature and dark survival leads to the theory that microalgae that fall to the bottom in cold and dark water layers have a higher chance of survival and seeding of new blooms when conditions are favorable (Sundqvist et al. 2018).

In the Nordic climate, the same microalgae that are obliged to survive in the absence of light must also deal with the high and prolonged irradiance during the summer. To endure the combination of low temperatures (0–5°C) and high light, which is highly deleterious for photosystem II (PSII), artic microalgae respond by a constitutive induction of non‐photochemical quenching (NPQ) (Robinson et al. 1997). NPQ is the dissipation of energy absorbed in excess of the capacity for utilization through heat, realized in plants through a carotenoid cycle during which violaxanthin is converted to antheraxanthin and then to zeaxanthin. To preclude the temperature‐imposed limitation on the enzymatic reactions, microalgae such as diatoms have a carotenoid cycle of just two compounds, diadinoxanthin and diatoxanthin. Moreover, arctic diatoms induce and maintain a large amount of the dissipating de‐epoxidated carotenoid (diatoxanthin) even at low light conditions, preventing damage to PSII. The presence of large amounts of carotenoids is accommodated in microalgae by proteins belonging to the Lhcx family of light harvesting proteins. Contrary to their names, these proteins do not participate in light harvesting but rather assist in the safe light dissipation as in *Phaeodactylum tricornutum* (Nymark et al. 2009, Bailleul et al. 2010), *Thalassiosira pseudonana* (Zhu and Green 2010) and *Fragilariopsis cylindrus* (Mock et al. 2017). Microalgae living on snow and ice deal with the excess solar and UV‐B irradiation by inducing a large pool of antioxidant compounds represented by lutein, α‐tocopherols and astaxanthin (Leya et al. 2009). Moreover, the silica frustules of diatoms confer protection against DNA damage by UV‐B irradiation (Aguirre et al. 2018). Compared to their arctic counterparts, light responses of temperate microalgae are not limited by the low temperature and are more complex, including a fast‐inducible and higher‐than‐plants NPQ (Ruban et al. 2004, Goss and Lepetit 2015), efficient clearance and repair of damaged PSII (Wu et al. 2012) and increased rate of CO_2_ fixation (Ni et al. 2017).

The eukaryotic microalgae dominating the marine environment in the Northern hemisphere are the diatoms (Armbrust 2009). Equally successful in other regions of the oceans, they contribute to about 20% of global carbon fixation and are key players in the biogeochemical cycling of nutrients in the ocean (Armbrust 2009, Smetacek 2018). The supremacy of diatoms in such dynamic environments is due to their special adaptations, including: (1) unique mechanisms for carbon, nitrate and phosphorus utilization (Armbrust et al. 2004, Kroth et al. 2008, Dyhrman et al. 2012, Hockin et al. 2012); (2) ability to withstand long periods of darkness while maintaining a functional photosynthetic apparatus (Nymark et al. 2013); (3) high and fast inducible NPQ (Kashino et al. 2002, Ruban et al. 2004, Lavaud and Kroth 2006, Taddei et al. 2018) and (4) survival for long periods of time in sediments until favorable conditions occur (Härnström et al. 2011).

## Exploring and exploiting the natural diversity of microalgae

The success of microalgae biotechnology builds on the choice of species, no matter the desired product. If a strain is not suitable for the purpose, any strategy to increase production of the compound of interest will have limited success. The most important species‐specific traits that need to be taken into account when considering microalgae for efficient cultivation are: growth rate, the content of the compound of interest, ease of harvesting. For large‐scale cultivation, the flexibility of the strain to environment and the resistance to contamination are essential. Microalgae strains that naturally out‐compete other strains and use the adverse environmental conditions to their advantage are an intuitive choice for industrial growth. Table 1 highlights Nordic microalgae species/strains used in a laboratory scale for biotechnological purposes.

**Table 1:** Overview of Nordic microalgae strains grown at laboratory scale for biotechnological purposes. The table provides information about the geographic origin, growth conditions, biomass, lipid and PUFA content. ASW, artificial sea water; DW, dry weight; DHA, docosahexaenoic acid; EPA, eicosapentaenoic acid; N.D., not determined; PBR, photobioreactor; PUFA, poly‐unsaturated fatty acids; TFA, total fatty acids; WM, Walne's medium; WW, waste water.

		Growth conditions								
Species (strain)	Geographic origin	Temp (°C)	Light intensity (μmol m^−2^ s^−1^)	Bubbling	Medium	Scale	Biomass (g l)	Lipids (% DW)	PUFA (% TFA)	References
*Phaeodactylum tricornutum* (*M26*)	Norwegian fjords	10–15	120–150	1% CO_2_	WM	280 ml	0.44	25.5%	13.3% EPA 1.41% DHA	Steinrücken et al. (2017)
*Phaeodactylum tricornutum (M28)*	Norwegian fjords	10–15	120–150	1% CO_2_	WM	280 ml	0.9	43%	7.33% EPA 0.6% DHA	Steinrücken et al. (2017)
*Phaeodactylum tricornutum (M28)*	Norwegian fjords	5–25	Outdoor light	Air + CO_2_	f/2 + high nutrient	35 l Green Wall Panels	0.4–2.5	10–15%	30% EPA	Steinrücken et al. (2018b)
*Attheya septentrionalis* (*M21*)	Arctic	10–15	120‐150	1% CO_2_	WM	280 ml	0.2	19%	24% EPA 3% DHA	Steinrücken et al. (2017)
*Attheya septentrionalis* (*M21*)	Arctic	10	50	1% CO_2_	WM	260 ml	0.28	27%	26%	Steinrücken et al. (2018a)
*Chlamydomonas sp*.	Svalbard	6	135‐230	2.5% CO_2_	f/2 + 3× NaNO_3_	350 ml PBR	3.4	28–39%	N.D.	Hulatt et al. (2017)
*Porosira glacialis*	Barents Sea	7	66	ND	f/10	300 l plexi columns	N.D.	6.39%	28% EPA 3% DHA	Artamonova et al. (2017a,b)
*Porosira glacialis*	Barents Sea	5	33	20–25% CO_2_	f/10	100 l plexi columns	N.D.	10.57%	23.6% EPA 5.75% DHA	Artamonova et al. (2017a,b)
*Skeletonema marinoi* (*SMTV1*)	Baltic Sea	15	300‐500	12–15% CO_2_	f/2	4.2 l polystyrene PBR	0.08	20%	N.D.	Olofsson et al. (2015)
*Skeletonema costatum*	Baltic Sea	4	100	Air	ASW	8 l polycarbonate bottles	N.D.	15.7%	N.D.	Schwenk et al. (2013)
*Scenedesmun sp*. (*UHCC0027*)	Baltic Sea	22	220	0.04–3% CO_2_	WW	24‐well plate	N.D.	23%	N.D.	Lynch et al. (2015)
*Scenedesmus* (*UHCC0027*)	Baltic Sea	6	225	ND	WW	24 l PBR	0.4	30%	N.D.	Jämsä et al. (2017)
*Coelastrella sp*.	North Sweden	25	100	Air	WW	80 ml multicultivator	1.46	30.8%	N.D.	Ferro et al. (2018a,b)
*Scenedesmus sp*.(B2‐2)	North Sweden	25	100	Air	WW	80 ml multicultivator	1.36	22.4%	N.D.	Ferro et al. (2018a,b)
		5					1.4	30%		
*Chlorella vulgaris* (*13‐1*)	North Sweden	25	100	Air	WW	80 ml multicultivator	1.15	34.1%	N.D.	Ferro et al. (2018a,b)
		5					1.2	26%		
*Desmodesmus* (*RUC‐2*)	North Sweden	25	100	Air	WW	80 ml multicultivator	0.9	30%	N.D.	Ferro et al. (2018a,b)
		5					0.7	21%		
*Nannochloropsis oculata* (UTEX LB2164)	Culture collection	20	50–200	0.3–2% CO_2_	WW	400 ml flat panel PBR	0.86	24.5%	22% EPA	Polishchuk et al. (2015)

### Marine microalgae

In the search for a strain with attractive biotechnological potential in the Nordic climate one should consider the reservoir of genetic diversity represented by marine algal blooms, which are observed annually in the North Atlantic and in the North Sea. The timing of the diatom blooms is usually at the beginning of spring, when phytoplankton growth is severely restricted due to limiting light and low temperature. At this time, nutrients accumulate in surface waters and as soon as light availability increases in the spring, the phytoplankton will exploit the abundant nutrients and multiply rapidly, producing blooms. Blooming microalgae strains, characterized by good growth in low temperature and limiting light, constitute a relevant ecological source for selection of strains for outdoor growth in the cold season (Saravanan and Godhe 2010, Kremp et al. 2012). The early spring blooms in the temperate and arctic marine waters are dominated by the diatom genera *Chaetoceros*, *Skeletonema*, *Thalassiosira* and *Fragilariopsis* (Degerlund and Eilertsen 2010). Diatoms tend to dominate blooms in spring, when surface waters are well mixed and are replaced by coccolitophores blooms later during spring, i.e. May, when surface waters are warmed up. The ubiquitous coccolitohophore *Emiliana huxleyi* dominates the summer blooms in the North Atlantic.

Nitrate, phosphorus and silica are the main nutrients limiting the duration and the density of microalgae blooms. The duration and the termination of an algal bloom are also affected by biotic factors such as grazers and viruses. A large virus infects the cosmopolitan coccolithophore *Emiliania huxleyi* causing lysis of blooms (Bratbak et al. 1993). Viral infection induces metabolic reprogramming of host cells, triggers lipidome remodeling and increase in the content of highly saturated TAGs (Malitsky et al. 2016). The virus‐mediated lysis of cells and TAGs release into the media could inspire a new strategy for TAGs harvest for algae‐based biodiesel production.

As an adaptation to low temperatures, microalgae increase the amount of PUFAs, such as eicosapentaenoic acid (EPA, 20:5) and docosahexaenoic acid (DHA, 22:6), in their membranes. These fatty acids are essential for human health by preventing cardiovascular and inflammatory diseases. A screening pipeline was employed at University of Bergen, Norway, on a collection of 150 microalgae clones isolated from various locations in the North Atlantic (60–80°N) (Steinrücken et al. 2017) with the aim of identifying strains with high biomass and high EPA and DHA levels. Three diatom strains showed potential for large‐scale cultivation at low (10°C) to moderate (15°C) temperatures for high EPA and DHA production. Two strains of the diatom *P. tricornutum* and one strain of *Attheya septentrionalis* showed high growth rates (≥ 0.7 divisions day^−1^) and increased EPA content in laboratory scale experiments (Table 1). When the two local strains of *P. tricornutum* were grown outdoors, in flat panel photobioreactors, during a 6 months period (May–October) they were outperformed in terms of EPA production by a commercial strain of *P. tricornutum* (Steinrücken et al. 2018b). Moreover, outdoor vs indoor (laboratory) cultivation had a profound effect on the fatty acid composition. The arctic strain of *Attheya septentrionalis* was grown in a factorial design experiment in which growth phase, salinity and irradiance were applied in different combinations to reveal the effect on EPA production (Steinrücken et al. 2018a). The highest EPA content (7% of dry weight) was obtained at lower salinity of the growth media (salinity of 22) and after 5 days in stationary phase.

The dependence of dark metabolism on the temperature observed in arctic and temperate microalgae can be exploited for biotechnological purposes. In different cultivation systems, the night period is a tax on the biomass accumulation during the day, with losses between 1% and 22% (Edmundson and Huesemann 2015). A study that investigated biomass loss during darkness found that four days of dark treatment led to an increase from 23% to 54% in lipid content in *P. tricornutum* (Bai et al. 2016). The increased lipid content was achieved through decrease in photosynthesis‐related machinery and nitrogen assimilation pathway, and increase in protein degradation and fatty acids biosynthesis pathways. This study shows the potential of using the low‐cost dark treatment to increase the lipid content in arctic/temperate diatoms.

Efficient year‐round outdoor algae cultivation at northern latitudes would require a microalgae strain that is able to perform equally well at low temperatures (during the winter) and at around 20°C, which is the average summer temperature (at the same location, e.g. Gothenburg, 57.7°N, 11.97°E). Moreover, if closed photobioreactors are used, the temperature inside can reach 40°C during the summer (Ekendahl et al. 2018). It is reasonable to assume that a strain fitted to all conditions is impossible to find. Another approach would be to use one winter (low‐temperature) strain and one summer (high‐temperature) strain. Strains of the marine diatom *Skeletonema marinoi* were isolated from the west coast of Sweden during two succeeding years at different seasons and maintained in a collection at University of Gothenburg, Sweden. The collection is currently undergoing a screening for strains that present robust growth and high biomass/lipid production when grown under simulated outdoor conditions specific for the climate of the west coast of Sweden (Cheregi, personal communication). The study aims for a rotational and year‐round strategy of outdoor algae cultivation. This approach has been successfully implemented in Japan (Matsumoto et al. 2017), by growing the cold‐tolerant diatom *Mayamea* sp. JPCC CTDA0820 during November–March and the oleaginous diatom *Fistulifera solaris* during April–October, in outdoor open‐ponds. Interestingly, the biomass productivities reached during the 6 months of the cold season, with average temperatures below 15°C, were very similar to productivities obtained in the warmer months. However, lipid productivities were slightly lower in the cold than in the warm season.

### Arctic microalgae

Most microalgae from the temperate zone that are used in biotechnology reach their maximal growth at temperatures of minimum 15°C, a condition that is fulfilled only during the summer months. Cultivation in other seasons requires heating of the system, which increases the production cost unless waste heat is used. Selection of species from arctic or alpine regions mitigates some of the cold negative effects on productivity (Varshney et al. 2015).

Microalgae strains from the ice‐covered regions of the Northern hemisphere (psychrophilic algae) overcome the adverse effects of low temperature on biochemical reactions by encoding enzymes with high catalytic efficiency and by increasing the level of membrane unsaturation (Hulatt et al. 2017). By increasing the content of PUFA such as EPA and DHA in their membranes, psychrophilic microalgae are exploitable sources of these valuable compounds. Green algae isolated from snow or solid substrates in Svalbard (78–79°N, 11°E) were used in a photobioreactor setup at 6°C to obtain high biomass rich in lipids (Table 1, Hulatt et al. 2017). The main factor influencing the high autotrophic productivity at this low temperature (∼0.6 g biomass l^−1^ day^−1^) seemed to be carbon limitation. Although CO_2_ solubility is increased in cold water, the gas–liquid mass transfer is lower, so that the addition of concentrated CO_2_ (1–2.5%) becomes essential for high biomass production (Hulatt et al. 2017). By comparing the results of this study with those obtained using a similar bioreactor design but at higher temperatures (25°C), the authors place their data in the broad context of 60 years compilation of photobioreactor cultivation experiments with a very encouraging message: depending on the strain choice and photobioreactor design, the productivity at low temperature is in the range of productivities obtained at warm temperatures.

In search for sustainable PUFA sources, arctic microalgae species from Norwegian fjords were cultivated in nutrient‐replete media and at temperatures relevant for the spring blooms in coastal waters of Norway (7°C) (Artamonova et al. 2017a). The obtained high EPA and palmitic acid content indicated that Northern diatoms have the potential to replace fish oil in aquaculture feeds. A *Porosira glacialis* strain isolated from a spring bloom in the Barents Sea grew well at low temperatures (5°C), increased its biomass and even its lipid content when grown with 20–25% CO_2_ (Table 1, Artamonova et al. 2017b).

The use of arctic diatom cultivation could be extended to production of bioactive compounds. Five diatoms species isolated from the North Atlantic and grown at 4–9°C showed bioactivity against diabetes, cancer, infection and had antioxidant properties (Ingebrigtsen et al. 2016). Cultivation of six diatom species isolated from spring blooms in the North Atlantic at two different temperatures (0.5 and 8.5°C) revealed that temperature not only determines the metabolic fingerprint but also modulates the chemical diversity. Growth at low temperature increases the chemical diversity, an effect that has to be taken into account when prospecting for bioactive compounds (Huseby et al. 2013).

### Brackish water microalgae

The second largest brackish water body in the world, the Baltic Sea, is also the scene of successive phytoplankton blooms: diatoms and dinoflagellates bloom in the spring and in the autumn and cyanobacteria during the summer. The most successful diatom species during the spring blooms is *Skeletonema marinoi*, that reaches up to 10^4^ cells ml^−1^ in the Kattegat region of the Baltic Sea (Saravanan and Godhe 2010). The same diatom is found in the spring blooms covering the central part of the Baltic basin, although the timing of the bloom is slightly delayed (Godhe et al. 2016). *Skeletonema* is a cosmopolitan genus (Kooistra et al. 2008) that has successfully colonized global coastal waters. Owing to its wide distribution, the genus has been considered for biotechnological purposes, e.g. lipid production for bioenergy and for fish feed (Bertozzini et al. 2013, Jiang et al. 2016, Pérez et al. 2017). A monoculture of *Skeletonema marinoi* and a natural microalgae community isolated from a spring bloom in the Baltic Sea were investigated for the purpose of CO_2_ remediation using flue gases from a cement factory (Olofsson et al. 2015). Both the monoculture and the natural community doubled the biomass when cultivated with flue gases containing ∼15% CO_2_ proving the potential of microalgae for CO_2_ remediation in coastal regions. To the best of our knowledge, this is the only published report investigating the potential of Baltic strains for bioremediation and biomass production. Schwenk et al. (2013) investigated the dependence of lipid content and composition on the growth stage, temperature and salinity in a brackish strain of *Skeletonema*, and showed that its lipid content increases with decreasing nitrogen in the cells (Table 1).

### Fresh water microalgae

Microalgae isolated from fresh water sources (rivers, lakes, sewage treatment plants) have mostly been investigated for wastewater treatment, but also for biomass accumulation and lipid production. Locally isolated microalgae and even microalgae‐bacteria consortia have been used with success at laboratory scale for removal of nitrate and phosphorus from wastewater and for production of biomass and lipids (Table 1, Lynch et al. 2015, Jämsä et al. 2017, Ferro et al. 2018a, b). The ‘indigenous’ species with best performance in wastewater belong to green algae, whereof *Scenedesmus*, *Desmodesmus* and *Chlorella* are among the most successful species.

In Finland, screening of locally isolated strains of cyanobacteria and eukaryotic microalgae for growth and lipid production in municipal wastewater (Lynch et al. 2015) identified a strain belonging to the *Scenedesmaceae* family (UHCC0027) as the best producer of biomass and lipids. The potential of this strain to grow under the Nordic climate was further investigated by Jämsä et al. (2017). Nitrogen removal from the waste‐water was strongly influenced by temperature, while phosphorus removal was improved by modifying the N:P ratio. Interestingly, the lipid content of the alga grown at cold temperatures (6°C) was about the same during the mid‐exponential and late‐exponential growth phase (∼ 30%), while at room temperature the lipid content was increased about 2.5‐fold during the late‐exponential growth (∼ 50%) compared with mid‐exponential phase.

Using microalgae for wastewater reclamation proved to be a successful concept even in the harsh climate of North Sweden. Sixty‐two microalgae strains isolated from fresh and wastewater sources around Umeå (Ferro et al. 2018a) were tested for growth in wastewater at 25°C at laboratory scale. In‐depth analysis identified eight strains of *Desmodesmus*, *Chlorella* and *Coelastrella* that could completely remove nitrogen and phosphorus from wastewater while accumulating more than 30% lipids. These eight local strains were also found highly efficient at removing lipophilic pharmaceuticals from growth medium, and some of them were even able to metabolize these compounds (Gojkovic et al. 2019). Three local strains from Northern Sweden performed better than a collection strain (isolated from South Sweden) when grown in wastewater at different stress conditions (Ferro et al. 2018b), in a laboratory scale experiment. The combination of 5°C and continuous light produced the same amounts of biomass as those obtained in standard conditions (25°C, continuous light) and most interesting, allowed complete nitrogen and phosphate removal from wastewater. Cultivation in dark stress (25°C, 3 h light) led to a substantial inhibition of growth rate and biomass production but nitrogen and phosphate removal were almost complete. Using these natural and local strains, wastewater remediation could be prolonged during the subarctic winter season.

Cultivation of microalgae for biomass would benefit economically if high‐value products such as carotenoids or long‐chain PUFA also can be produced in the process of nitrogen and phosphorus removal from waste water. Interestingly, the marine microalga *Nannochloropsis oculata* could grow in waste water from a paper factory in Finland (Polishchuk et al. 2015) and produced EPA with the same productivity as in artificial seawater.

## Scaling up microalgal cultivation in the Nordic climate for testing and commercialization

A low‐cost and energy‐efficient microalgae cultivation system is recognized as one of the great challenges for a productive and profitable business. Points of concern include types of equipment, techniques for efficient supply of necessary light and/or nutrients including carbon and harvesting methods. Globally, cultivation is performed in different types of equipment: open raceways or other types of ponds with or without covers/greenhouses, (photo‐)bioreactors exemplified by flat panels and tubular reactors (Chen et al. 2011) as well as surface structures for attached microalgae growth. Optimization efforts aim at increased biomass and/or lipid productivity, improved mixing of gas and nutrients, maximal effect of light supply (if not cultivating heterotrophically) and cheap efficient harvesting methods.

Energy efficiency during cultivation in the Nordic climate was shown for the first time using a once‐a‐year harvesting strategy in so‐called Tethys ponds (Ekendahl et al. 2018). The demand for high productivity is usually contradictory to energy efficiency. For bulk production purposes, one step forward to make these end points meet is to maximize the efficiency of harvesting using bio flocculation combined with an optimized sedimentation speed before dewatering, taking advantage of the natural properties of microalgae, such as morphology, size and surface characteristics. Optimization of speed should prevent degradation of the biomass as far as possible, and this technical challenge should also focus on each specific intended product and quality of the biomass (Barros et al. 2015).

### Use of wastewater, waste heat and waste CO_2_

To reach energetic and economic profitability, not only the cultivation technique but also cheap and sustainable supplies of water and nutrients are of critical importance. The use of potential waste resources such as nutrient‐rich wastewater, industrial emissions of flue gas containing CO_2_ and waste heat of low grade for usage in colder climate zones is essential. Municipal wastewaters are usually rich in the essential nutrients such as nitrogen and phosphorus needed for efficient cultivation. Other types of wastewaters are available from industry but often contain low levels of the major nutrients. On the other hand, they may be useless for district heating purposes but warm enough for heating adapted cultivation systems or greenhouses. Industrial flue gas emissions contain CO_2_, NO_x_, SO_x_, N_2_, H_2_O, C_x_H_y_, O_2_, CO and possibly H_2_S as well as particles, heavy metals and halogen acids, and are hot (Van Den Hende et al. 2012). This heat may also be used to some extent. The gas is often filtered and cooled before reaching a cultivation site. The effect of particles and metals on algae growth has scarcely been investigated and can present positive effects in lower concentrations, toxicity problems at higher concentrations as well as sequestration opportunities for an algae cultivation site (Napan et al. 2015).

In Northern Europe, there have been many projects investigating the growth and performance of microalgae in waste resources at laboratory or small pilot scale outdoors, performed by universities and research institutes often in cooperation with industry. In addition, a handful of microalgae production companies are currently running at commercial scale in Northern Europe. Below we focus on companies that have either reached at least near commercial status or have cultivated microalgae outdoors at pilot scale for more than a year with published scientific results. These companies cultivate microalgae for high‐value products in relatively expensive photobioreactors. Examples include AstaReal AB producing astaxanthin from *Haematococcus pluvialis* cultivated in specifically designed stainless steel photobioreactors; Ostrea Aquaculture using indoor tubular photobioreactors with artificial light to grow marine diatoms (*Chaetoceros muelleri*, *Chaetoceros calcitrans*, *Skeletonema costatum*, *P. tricornutum*) and flagellates (*Tisochrysis lutea*, *Rhodomonas salina*, *Tetraselmis suecica*) for oyster larvae feed; and Simris Alg AB cultivating microalgae for PUFA and fish oil using tubular photobioreactors in greenhouses. Such companies do not generally use waste resources for their cultivation due to the high‐quality demand on the products. In Iceland, however, flue gas from a geothermal power plant is filtered to reduce the concentration of H_2_S, and thereafter used for cultivation of local microalgae in geothermal brine, consisting of a mixture of one‐third freshwater and two‐thirds marine water with high silica content supplied with additional nutrients. Geothermal electricity is used for heating. Among diatoms and other microalgae, the coccoid cyanobacterial species *Cyanobacterium aponinum* is grown in tubular photobioreactors at the Blue Lagoon for skin care products (Arnardottir et al. 2015).

Several pilot scale cultivations at industrial locations in the Nordic countries have been or are currently being performed. Common to all these cultivation sites is the use of waste resources for different remediation purposes, often combined with studies on production of biomass for bio‐based bulk compounds, mainly lipids, carbohydrates and proteins. The Tethys cultivation ponds discussed above was tested outdoors in natural light at the Nordic Paper Bäckhammar AB pulp and paper mill factory in south‐central Sweden for 3 years. Heat from the wastewater treatment plant at the factory and flue gas from the soda boiler was used in the study. The freshwater green algae strain *Tetradesmus obliquus* strain UTEX 417 (formerly *Scenedesmus dimorphus*) originally isolated from Southern Sweden was inoculated from start but it was later shown that local consortia dominated by *Chlorella*‐like microalgae soon developed in the Tethys ponds and grew well in the flue gas. Green algae of the *Monoraphidium*‐type dominated in a nearby raceway pond cultivated simultaneously. The study aimed for lipid production but most importantly showed that the special cultivation technique with harvesting only once a year was energy efficient (Ekendahl et al. 2018). The Tethys ponds have been developed further and are currently being used for remediation of CO_2_ and lead at the Boliden Bergsöe smelters in Skåne, Sweden (Strömberg, unpublished data).

At the waste incineration heat and power plant Umeå Energi AB in Northern Sweden, cultivation with a mixture of wild local freshwater green microalgae strains is performed outdoors in natural light in raceway ponds or in non‐heated greenhouses using waste water from the local municipality as well as flue gas from the power plant. Starting with a strain of *Tetradesmus*, local communities evolved and *Dictyosphaerium* was the dominating genera after 3 years of cultivation. A potential for reduction of pharmaceuticals was demonstrated (Gentili and Fick 2017). Removal of nitrogen and phosphorus was shown to be efficient using a mixture of wastewaters from the municipality, a dairy farm and a pulp and paper industry combined with flue gas from the heat and power plant. The green alga strain *Selenastrum minutum* showed the highest production of biomass and lipid content in this test (Gentili 2014). Co‐digestion of natural freshwater green algae dominated by *Scenedesmus* with municipal sewage sludge was also tested with the flue gas and showed an increased biomethane potential in mesophilic but not in thermophilic conditions (Olsson et al. 2014). Water effluent from a local biogas plant improved the nutritional content of a *Nannochloropsis salina* strain when grown in a 4000 l flat panel photobioreactor in the conditions of Danish winter (Safafar et al. 2016).

Waste heat and flue gas containing CO_2_, NO_x_, Si and inorganic nutrients from the ferrosilicon producer Finnfjord AS near Tromsö in Northern Norway is used for cultivation of the large arctic marine diatom *Coscinodiscus*. The large size is used for efficient settling of cells and to maintain good light penetration in the 300 000 l (world's largest) closed tank used for cultivation. The main aims are CO_2_ neutrality for the factory and production of bioactive compounds, PUFA and essential amino acids mainly for fish fodder. They have plans to expand to industrial scale (Hans C. Eilertsen, personal communication).

Municipal and industry wastewater are used at the Kungshamn (near Gothenburg) facility of the Swedish Algae Factory to cultivate microalgae and produce biomass for fish feed and organic fertilizer. The wastewater is used in a circular economy concept, where no waste is generated and the biomass utilizations are diverse. Relying on the natural properties of diatoms adapted to low light, the nanoporous silica shell of cultivated microalgae is used to coat solar panels and by that increase their efficiency with 4%. The nanoporous silica also confers UV‐B protection extending microalgae shell's use for UV‐resistant paints, plastics and cosmetics.

A national algae pilot plant has also been started at Mongstad, near Bergen in western Norway. Here the consortium CO2Bio AS (2018) (https://www.co2bio.no/) aims for production of biomass rich in PUFA in a 200 m^2^ greenhouse with tubular photobioreactors supplied with sea water and CO_2_ from the Technology Center in Mongstad.

## Future perspectives

Cultivation of microalgae for biofuels and chemicals has the potential to become a sustainable alternative to fossil fuels and traditional crops. The intense research and progress made even in countries with climate conditions less friendly for outdoor cultivation, predicts a future in which microalgae share in global production will be significantly higher. The biomass and lipid productions obtained by cultivating Nordic microalgae strains (Table 1) prove that year‐round microalgae cultivation in a low temperature environment can be achieved if local strains adapted to the local climate are used.

The characteristic climate in Nordic countries, with highly variable temperature and light conditions over the year, presents specific problems regarding the use of large‐scale cultivation techniques year round. Examples include development of harvesting methods to take advantage of these variable conditions and yearly rotation of local strains adapted to specific seasons. Nordic countries also have a surplus of low‐grade waste heat, which may not be available or seen as a resource in warmer countries, where overheating might instead be a large problem. Studies also show that the productivity and quality of lipids (content of unsaturated fatty acids) can change with seasons (i.e. Olofsson et al. (2014)), that might become beneficial compared to production at latitudes without variable seasons.

Sustainable large‐scale production of biomass from microalgae will require access to waste resources, such as sources of elevated concentrations of CO_2_ and inorganic nutrients, with low‐grade waste heat as an option to extend the growth season. Such access implies localization within effective transport distance of industries, power plants and areas with higher population density. Implementation within a near future will probably have a primary, or at least equal, focus on remediation of waste resources and on production of biomass for fuels or chemicals. A change in focus towards production of biomass as primary purpose will likely follow as society and industries shift to a higher ratio of bio‐based raw materials and the direct as well as indirect costs of environmental impact increase. The lower overall population density in Nordic countries as well as access to large bodies of water makes implementation of large‐scale microalgae cultivation feasible without competition with food production and arable land. Large collaborative consortia between the Nordic countries, such as NordAqua, promote interchange and cooperation in the field of biomass production and will lead to improved and diversified industrial applications of micro‐ and macroalgae in Nordic environments.

Despite the large biodiversity of marine microalgae species and the almost unlimited availability of sea water, there is a shortage of commercial marine strains. Culture collections of native microalgae strains present the excellent opportunity for exploring their potential for biotechnology. While performing the task of selecting high producing and robust strains, the availability of rapid and reliable methods for screening is also of outmost importance. Nature‐inspired strategies and the use of genetic engineering can be used to improve the quantity and the quality of biomass. The doubling of the lipid content in *Nannochloropsis*, through genome editing using CRISPR‐Cas9 (Ajjawi et al. 2017) paves the way for targeted mutagenesis to enhance synthesis of the desired compound, increase tolerance to environment and also create special traits such as flocculation, secretion of compounds or autolysis.

## Author contributions

O.C. and S.E. wrote the manuscript with input from all the co‐authors. All authors provided critical feedback that shaped the final form of the manuscript.
